# Do fragments and glycosylated isoforms of alpha-1-antitrypsin in CSF mirror spinal pathophysiological mechanisms in chronic peripheral neuropathic pain? An exploratory, discovery phase study

**DOI:** 10.1186/s12883-018-1116-2

**Published:** 2018-08-16

**Authors:** Emmanuel Bäckryd, Sofia Edström, Björn Gerdle, Bijar Ghafouri

**Affiliations:** Pain and Rehabilitation Center, and Department of Medical and Health Sciences, Linköping University, Linköping, Sweden

**Keywords:** Alpha-1-antitrypsin, Cerebrospinal fluid, Neuropathic, Pain, Pathophysiology

## Abstract

**Background:**

Post-translational modifications (PTMs) generate a tremendous protein diversity from the ~ 20,000 protein-coding genes of the human genome. In chronic pain conditions, exposure to pathological processes in the central nervous system could lead to disease-specific PTMs detectable in the cerebrospinal fluid (CSF). In a previous hypothesis-generating study, we reported that seven out of 260 CSF proteins highly discriminated between neuropathic pain patients and healthy controls: one isoform of angiotensinogen (AG), two isoforms of alpha-1-antitrypsin (AT), three isoforms of haptoglobin (HG), and one isoform of pigment epithelium-derived factor (PEDF). The present study had three aims: (1) To examine the multivariate inter-correlations between all identified isoforms of these seven proteins; (2) Based on the results of the first aim, to characterize PTMs in a subset of interesting proteins; (3) To regress clinical pain data using the 260 proteins as predictors, thereby testing the hypothesis that the above-mentioned seven discriminating proteins and/or the characterized isoforms/fragments of aim (2) would be among the proteins having the highest predictive power for clinical pain data.

**Methods:**

CSF samples from 11 neuropathic pain patients and 11 healthy controls were used for biochemical analysis of protein isoforms. PTM characterization was performed using enzymatic reaction assay and mass spectrometry. Multivariate data analysis (principal component analysis and orthogonal partial least square regression) was applied on the quantified protein isoforms.

**Results:**

We identified 5 isoforms of AG, 18 isoforms of AT, 5 isoforms of HG, and 5 isoforms of PEDF. Fragments and glycosylated isoforms of AT were studied in depth. When regressing the pain intensity data of patients, three isoforms of AT, two isoforms of PEDF, and one isoform of angiotensinogen “reappeared” as major results, i.e., they were major findings both when comparing patients with healthy controls and when regressing pain intensity in patients.

**Conclusions:**

Altered levels of fragments and/or glycosylated isoforms of alpha-1-antitrypsin might mirror pathophysiological processes in the spinal cord of neuropathic pain patients. In particular, we suggest that a putative disease-specific combination of the levels of two different N-truncated fragments of alpha-1-antitrypsin might be interesting for future CSF and/or plasma biomarker investigations in chronic neuropathic pain.

**Electronic supplementary material:**

The online version of this article (10.1186/s12883-018-1116-2) contains supplementary material, which is available to authorized users.

## Background

A substantial part of our knowledge about the pathophysiology of pain has been acquired through animal experiments. Although there are obvious similarities between species, there are also differences, and translating evidence from animals to humans in this field is far from trivial [1]. The quest for *human* biomarkers, which would mirror the pathophysiology of different chronic pain conditions, must be understood against this background. Biomarkers would be useful for diagnosis and prognosis of different pain conditions, for the evaluation of treatment response, and for the development of drugs; they could also serve as surrogate endpoints (i.e., as substitutes for clinical endpoints) [2].

Post-translational modifications (PTMs) generate a tremendous protein diversity from the ~ 20,000 protein-coding genes of the human genome, the complexity of the proteome being several orders of magnitude greater than the coding capacity of the genome [3–5]. After the genome, mapping the proteome is next in turn [6]. Whereas the genome is constant, the proteome is continuously modulated by genome-environment interactions [7, 8]. PTMs modulate enzyme activity, protein turnover and localization, protein-protein interactions, various signaling cascades, DNA repair, and cell division [5].

Glycosylation, i.e. when a carbohydrate is attached to a protein, is one type of PTM [5]. The glycosylation form of a protein can be altered significantly because of changes in cellular pathways and processes resulting from inflammatory conditions, neurodegeneration, or cancer [9]. These potentially detectable protein modifications may lead to the discovery of specific and sensitive biomarkers [10]. Protein fragments, i.e. proteins that have been truncated either at the N- or C-terminal end of the amino acid sequence, are also potential specific biomarkers [11]. Indeed, in the context of dementia, the term “protein fragmentology” has been used [12], as has the term “degradome research” [13]. In the pain field, such a well-known neuropeptide as Substance P has biologically active and detectable fragments [14].

In chronic pain conditions, exposure to pathological processes in the central nervous system (CNS) could perhaps lead to a disease-specific fragmentation process detectable in the cerebrospinal fluid (CSF). Protein fragments are also interesting because their smaller size would enable them to cross the blood-brain barrier (BBB) easier than full-length proteins, and hence fragments would probably be easier to detect in blood [12].

Neuropathic pain is defined as pain caused by a lesion or disease in the somatosensory nervous system [15]. In a previous comparative two-dimensional gel electrophoresis study [16], we described seven CSF proteins highly discriminating between neuropathic pain patients and healthy controls. These seven proteins were one isoform of angiotensinogen (AG), two isoforms of alpha-1-antitrypsin (AT), three isoforms of haptoglobin (HG), and one isoform of pigment epithelium-derived factor (PEDF). The three aims of the present exploratory, discovery phase study [17] were:

1. To examine the multivariate inter-correlations between all identified isoforms of these seven proteins, using multivariate data analysis by projection (MVDA) [18, 19]. The focus here was not on discriminant analysis but rather on the internal correlation structure between these isoforms in health vs. neuropathic pain. Our hypothesis was that neuropathic pain is associated with an altered correlation structure between the different isoforms of a particular protein, compared to healthy controls.
2. Based on the results of the first aim above, to characterize PTMs in a subset of interesting proteins. Because protein fragments seem especially promising as biomarkers (their generation by disease-specific processes could reduce the overlap between diagnostic groups) [12], special attention was given to fragmented proteins [11].
3. Returning to MVDA and focusing on the patients, to regress clinical pain parameters (pain intensity and pain duration), using all the proteomic data (260 proteins) of our previous study as predictor variables [16]. We wanted to test the hypothesis that the above-mentioned seven discriminating proteins and/or the characterized isoforms/fragments of aim (2) above would be among the proteins having the highest predictive power for either pain intensity or pain duration.

Hence, the purpose of the study was not to conduct clinical biomarker research at the validation stage; instead, this was a pre-clinical exploratory study in the early discovery stage [17, 20].

## Methods

### Patients

The patients have been described extensively in a previous paper [16]. All pain patients included in this study were participating in a clinical trial of intrathecal bolus injections of the analgesic ziconotide [21]. Inclusion criteria were: 1) patient, at least 18 years of age, suffering from chronic (≥6 months) neuropathic pain due to trauma or surgery, who had failed on conventional pharmacological treatment; 2) average Visual Analogue Scale chronic Pain Intensity (VASPI) last week ≥40 mm [22]; 3) patient capable of judgment, i.e. able to understand information regarding the drug, the mode of administration and evaluation of efficacy and side effects; 4) signed informed consent.

After informed consent, the following data were registered: basic demographic data; pain diagnosis; pain duration; present and past medical history; concomitant medication. A medical examination was performed. All patients had at least probable post-traumatic/post-surgical neuropathic pain according to the criteria published by Treede et al. [23], and all were or had been candidates for Spinal Cord Stimulation. Detailed patient characteristics have been published elsewhere [16, 21]. After CSF sampling, the patient received an intrathecal bolus injection of ziconotide according to the protocol of the clinical trial.

For an overview of patients vs healthy controls, see Table 1.

**Table 1:** Overview of patients and healthy controls

Variables	Patients (*n* = 11)	Healthy controls (*n* = 11)	Statistics *p*-value
Age (years)	58 (35–75)	23 (20–28)	< 0.001*
Sex (% female)	55%	55%	1.0
Body Mass Index (kg/m^2^)	24.7 (20.2–30.0)	22.6 (20.8–26.5)	0.065
Pain duration (months)	65 (30–180)	0	< 0.001*
Pain intensity (0–100 mm)^a^	72 (40–87)	0	< 0.001*
Opioid dose^b^ (mg/day)	0 (0–480)	0	0.076
On opioids (%)	45%	0%	0.035*
On tricyclics or duloxetine (%)	36%	0%	0.090
On gabapentinoids (%)	36%	0%	0.090
On paracetamol^c^ (%)	45%	0%	0.035*
On NSAID^c^ (%)	18%	0%	0.476

Data are presented as median (range) or percentages. Furthest to the right is the result of the statistical comparisons between patients and healthy controls. * denotes significant group difference

Notes:

^a^: At inclusion, patients were asked to grade their average pain intensity for last week on a Visual Analogue Scale 0–100 mm, whereas the pain status of healthy controls was investigated by an extensive structured interview. All controls were free of pain

^b^: In oral morphine equivalents

^c^: Excluding treatment “as needed”. NSAID: Non-Steroidal Anti-Inflammatory Drug

### Healthy controls

Healthy controls were recruited by local advertisement at the Faculty of Health Sciences, Linköping University, Sweden, and by contacting healthy subjects from earlier studies. After informed consent, a structured interview was conducted to ensure the absence of any significant medical condition. The following areas were specifically assessed in the interview: earlier major trauma; back, joints, muscles or skeletal disease; heart or vascular disease; lung or bronchial disease; psychiatric symptoms; neurological, ear or eye disease; digestive tract disease; kidney, urinary or genital disease; skin disease; tumor or cancer; endocrine disease; hematological disease; birth defects; other disease, disability or allergy. Moreover, the presence of a known bleeding disorder was specifically inquired for.

The absence of a chronic pain condition was ensured by a structured questionnaire covering sociodemographic data, presence of pain now, location of pain now, generalization of pain, presence of intermittent pain, duration of persistent pain. The questionnaire also covered anxiety and depressive symptomatology using Hospital Anxiety and Depression Scale [24], coping aspects (i.e., catastrophizing) using Pain Catastrophizing Scale [25], and health-related quality of life aspects using Short Form-36 (SF-36) [26], in order to ensure that the controls were healthy. Subjects were also given the possibility to make a pain drawing about Pain Now, Pain at worst and Pain at best. Musculoskeletal pain was more deeply assessed by VASPI last month for 9 specific anatomical locations: neck; shoulders; arms; hands; upper back; lower back; hips; knees; feet. Concomitant medicines were registered. A medical examination was performed, including assessment for fibromyalgia tender points.

### Procedures

For every subject in this study, intrathecal access was obtained by lumbar puncture with a 27 GA pencil-point Whitacre needle (BD Medical, Franklin Lakes, New Jersey, USA) and a 10 ml sample of CSF was drawn in five numbered syringes of 2 ml each. Each sample was immediately cooled on ice and transported to the Painomics® laboratory, Linköping University Hospital, centrifuged and divided in aliquots and stored at − 70^°^C until analysis.

### Biochemical analyses

The comparative proteomic study between patients and healthy controls was performed as described in our previously study [16]. Briefly, 100 μg of depleted CSF proteins from each subject (11 patients and 11 healthy controls) were separated by 2-DE, visualized by silver staining and the protein patterns were digitalized and quantified using CCD camera (VersaDoc™ Imaging system 4000 MP, Bio-Rad) in combination with a computerized imaging 12-bit system designed for evaluations of 2-DE patterns (PDQuest 8.0.1 Bio-Rad). The different gel images were evaluated and protein spots were quantified according to spot optical densities (SOD). The generated SODs were evaluated for significant differences between the groups.

For the characterization of the different protein isoforms, a pooled CSF sample from patients and a pooled sample from healthy subjects were used. The samples were desalted, lyophilized and dissolved in urea sample buffer solution, as has been described in detail elsewhere [16]. Protein concentration was determined before and after desalting step using Bradford assay [16]. To examine N-glycosylation, 300 μg of CSF proteins were incubated in presence or absence of an *N*-glycosidase PNGase F (Sigma Aldrich) at 37 °C overnight using conditions recommended by the supplier and as has been described in detail elsewhere [27]. The proteins were then analyzed by 2-DE.

The interesting protein spots were excised from the gels, trypsinated and identified by liquid chromatography tandem mass spectrometry (LC-MS/MS) using Linear Trap Quadropole (LTQ) Orbitrap Velos Pro hybrid (Thermo Fisher Scientific) in conjunction with nano flow HPLC system (EASY-Nlc II, Thermo Fisher Scientific). Data processing of the spectra was performed using MaxQuant software, and the generated mass list was searched against SwissProt human protein sequence database as previously described [16]. When identifying fragments of proteins, the position of the matched peptides within the theoretical sequence of the protein were computed using the proteomic tool Compute *pI/MW* (http://www.expasy.org/proteomics). The calculated pI*/MW* of the fragment was controlled to be in agreement with the apparent mass and *pI* on the 2D-gel.

### Statistics

Traditional univariate statistical methods can quantify level changes of individual substances but disregard interrelationships between them and thereby ignore system-wide aspects. Therefore, we used SIMCA version 13.0 (Umetrics AB, Umeå, Sweden) for MVDA computations. Conceptually, imagine a multidimensional space where each protein is a dimension (“k” dimensions). Each subject (patient or control) will be a point in this k-dimensional space. Due to a combination of technological development (rendering high “k”) and practical/economic constraints (leading to a low number of subjects “n”), todays data tables in the omics field often have a low subjects-to-variables ratio (n < <<k). Classical regression techniques like multiple linear regression (MLR) or logistic regression (LR), which were developed in the early days of the twentieth century, are not suited for such high-dimensional and multi-collinear data. Hence, todays data table often break one of the underlying assumption behind MLR and LR, namely that the predictor (X) variables are fairly independent. MLR and LR also assume that a high subject-to-variables ratio is present (e.g., > 5), and they have difficulties coping with missing data. Due to the above-mentioned drawbacks of classical regression techniques (with regression coefficients becoming unstable and their interpretability breaking down), the modern MVDA methods of Principal Component Analysis (PCA) and Orthogonal Partial Least Squares (OPLS) regression were used instead. PCA and OPLS can handle subject-to-variables ratios < 1, and they cope well with both multi-collinearity and missing data. OPLS is a recent, easier-to-interpret modification of Partial Least Squares (PLS). The MVDA workflow and the reporting of parameters necessary for evaluating model quality were in accordance with the paper published by Wheelock & Wheelock [19]. For all MVDA analyses, data were log-transformed when needed (using the SIMCA function “auto transform selected variables as appropriate”) and scaling to unit variance was applied [18, 19].

For Aim 1 we used PCA, which is the foundation of all latent variable projection methods, separately for patients (*n* = 11) and healthy controls (*n* = 11), focusing on all the isoforms of the seven proteins mentioned in the introduction. Each isoform had previously been quantified by SOD [16]. In a multivariate data set, important information can be found in the correlation structure of the whole data set, i.e. in the inter-correlations between all the variables taken together as a whole. PCA entails the definition of a few latent variables that describe the underlying structure in the data. The latent variables (called principal components, PC) are uncorrelated to each other, and they summarize and simplify the data, separating information from noise and enabling to find relevant patterns in the data. Optimal model dimensionality (i.e. number of PCs) is determined by cross-validation, which is a practical and reliable way to test the significance of a PCA model. This is default in SIMCA. Hence, PCA can be viewed as a form of multivariate correlation analysis. PCA also enables the identification of multivariate outliers and deviant subgroup, as assessed by Hotelling’s T2 statistic (T^2^ Critical 95%) and by distance to model in X-space (DModX). The R^2^ value indicates how well the model explains the dataset, and cross-validated Q^2^ is a measure of the predictive power of the model. If R^2^ is substantially greater than Q^2^ (a difference > 0.3 is mentioned in the literature) [18], the robustness of the model is poor, suggesting overfitting [19].

A PC relates to each original variable by a loading, which has a value between − 1 and + 1. Variables with high loadings (ignoring the sign) are considered to be of large or moderate importance for the PC under consideration. Hence, PCA is a data visualization technique that models the correlation structure of a dataset, presenting the relationship between variables in a loading plot. On a loading plot, variables close to each other are positively correlated, and variables that are unimportant for the model are found around the origin of the plot (i.e., variables with loadings near zero do not contribute to the model) [18].

For Aim 3, OPLS was used to regress (predict) two clinical variables in patients: VASPI last week and pain duration. Hence, the outcome variable (Y) was one of these two clinical variables, whereas the predictor variables (X:s) where the relative quantification of 260 proteins by SOD in accordance with our previous study [16]. Concerning optimal model dimensionality (i.e. the number of latent variables) and R2/Q2, see above.

In OPLS, the importance of each variable for the model can be measured as a Variable Influence on Projection (VIP) value. This indicates the relevance of each X-variable pooled over all dimensions and Y-variables – the group of variables that best explain Y. Variables with VIP ≥ 1.0 and having a 95% confidence interval not including zero are usually considered significant, but in this study VIP≥ 1.5 was used. The direction of the relationship (positive or negative) was determined by sign of the corresponding loading.

For traditional univariate statistics, all computations were made using IBM® SPSS® Statistics version 23. Spearman’s rho correlation coefficient was used for bivariate correlation analysis, and Mann-Whitney U test or Fisher’s exact test were used for comparing groups (for continuous and categorical data, respectively). A two-sided significance level of 0.05 was chosen.

## Results

### Correlation structure in patients vs. controls (aim 1)

**Fig. 1:**
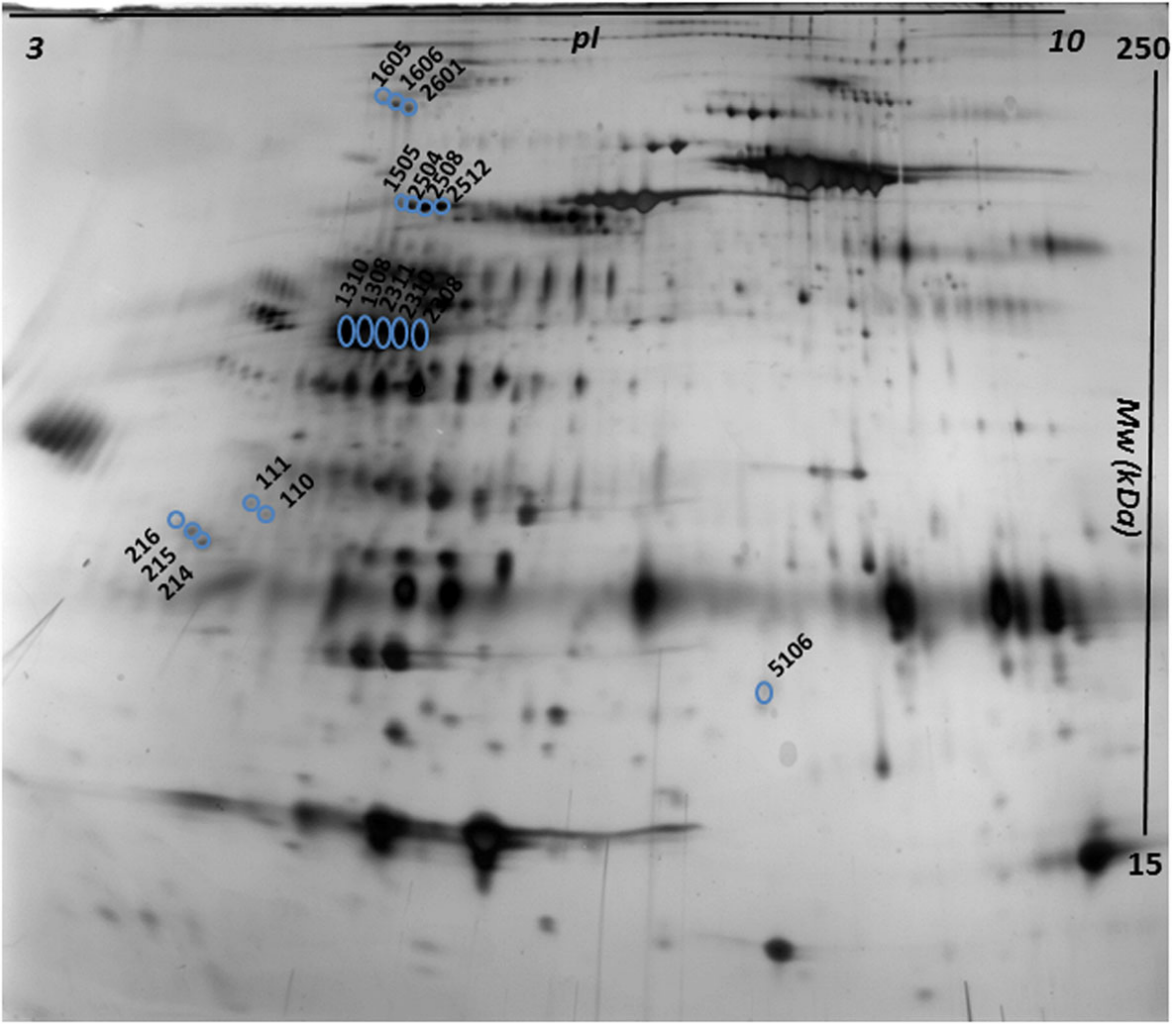
Typical cerebrospinal fluid two-dimensional electrophoresis gel, highlighting the 18 isoforms alpha-1-antitrypsin with their spot number. Proteins separate according to pI (range 3–10) and according to Mw (range 15–250 kDa)

We identified 5 isoforms of AG, 18 isoforms of AT, 5 isoforms of HG, and 5 isoforms of PEDF – amounting to a total of 33 proteins. Hence, we generated a SIMCA data table consisting of 22 individuals (rows) and spot optical densities from 33 proteins (columns). To enable quick identification when looking at loading plots (see below), AG, AT, HG, or PEDF was added to the original spot number. Moreover, on basis of their location on the gels, five groups of AT were identified, which were referred to by Roman numeral I-V; AT5106 did not belong to any group (Fig. 1). Because of the large number of missing values in AT group V (3 isoforms with missing values in 68%, 68% and 63% of cases, respectively), proteins from that group were not included in the analysis of Aim 1. Hence, the statistical models described below were based on 30 protein variables.

**Fig. 2:**
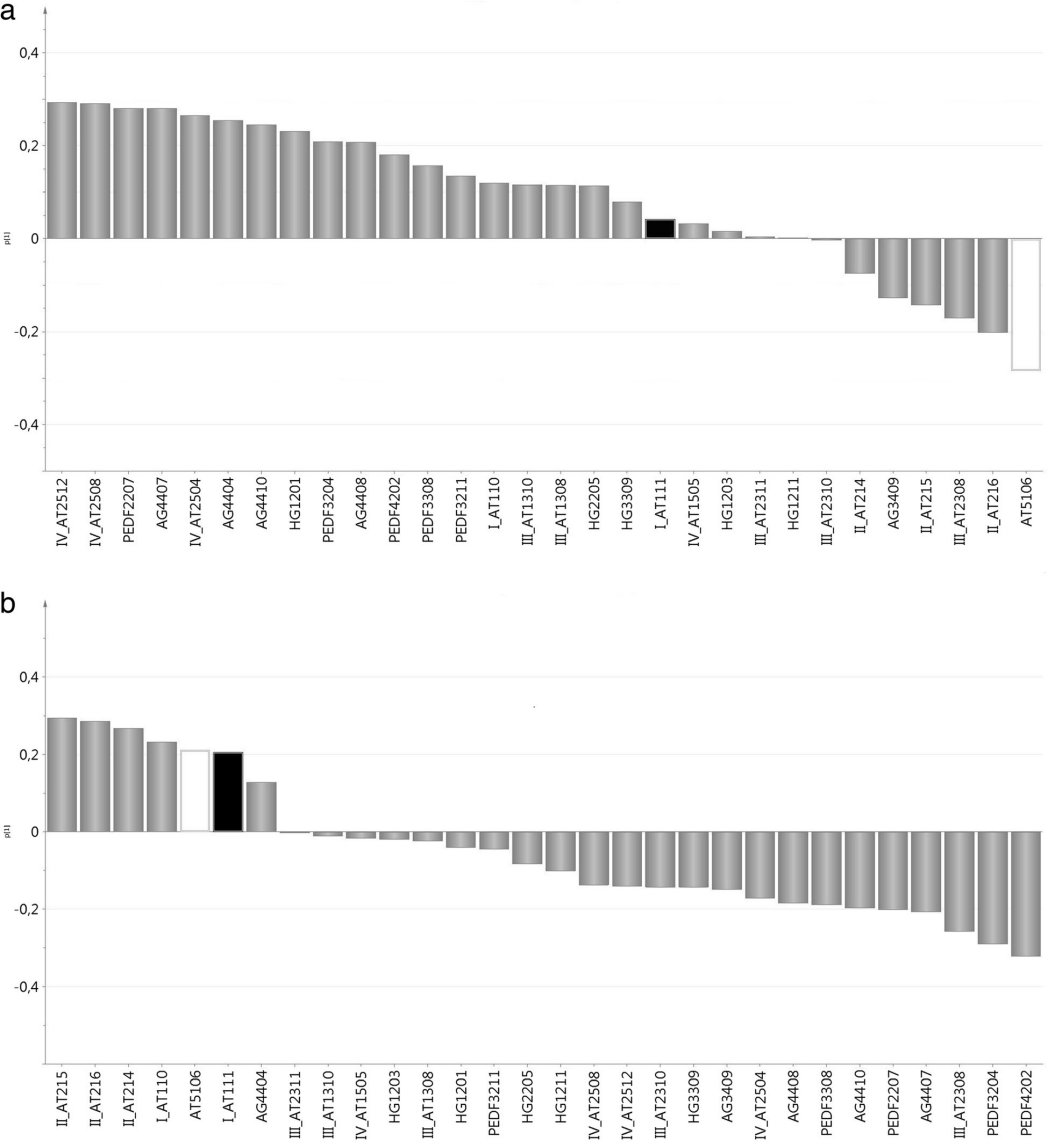
Loadings column plot for healthy controls (**a**) (*n* = 11) and neuropathic pain patients (**b**) (*n* = 11). Each column represents the value of the loading p [1] of that particular protein in the principal component analysis (PCA) model. The columns of the two fragments of alpha-1-antitrypsin (AT) that the present paper focuses on (I_AT111 and AT5106) are depicted in black and white, respectively. Other protein abbreviations are angiotensinogen (AG), haptoglobin (HG), and pigment epithelium-derived factor (PEDF). The number after each protein name abbreviation corresponds to the spot number

First, an unsupervised PCA model for healthy controls (*n* = 11) was computed. The model had one PC (R^2^ = 0.31, Q^2^ = 0.12). No multivariate outliers were found. The loadings column plot of the model is depicted in Fig. 2a. Then, an unsupervised PCA model for patients was computed (*n* = 11). The model had one PC (R^2^ = 0.29, Q^2^ = 0.02). No multivariate outliers were found. The loadings column plot of the model is depicted in Fig. 2b. Then, the two loadings column plots were compared (Fig. 2a and b), focusing on the seven proteins with the highest discriminatory power between patients and healthy controls according to our previous study [16], namely AG3409, AT5106, IV_AT1505, HG1211, HG1203, HG2205, and PEDF3308:

- **AG3409:** In healthy controls, AG3409 is separated from the four other isoforms of AG, and these four isoforms inter-correlated positively, ie the loading values (p [1]) were similar. This correlation structure is disrupted in patients in the sense that, in patients, it is AG4404 that is separated from the four other isoforms of AG.
- **AT5106:** In Fig. 2b, the p [1] value of I_AT111 (black column) is almost the same as that of AT5106 (white column), i.e. these two proteins inter-correlated positively in patients. In healthy controls (Fig. 2a), this was not the case. We have previously shown that AT5106 was downregulated in patients, whereas I_AT111 (although not being one of the seven highest discriminating proteins) was upregulated [16]. Hence, in patients, a down-regulated isoform of AT correlated by PCA to an up-regulated isoform of AT. However, looking at these two proteins with traditional bivariate correlation (i.e., not multivariate PCA), there was no statistically significant association between them, neither in patients nor in healthy controls.
- **IV_AT1505:** In both patients and healthy controls, IV_AT1505 is close to zero, meaning that this isoform does not contribute much to the two PCA models. IV_AT1505 also remains fairly isolated from the other isoforms of group IV. The remaining isoforms of group IV of AT positively inter-correlate in a similar way in both health and disease.
- **HG1211, HG1203, HG2205**: No clear correlation structure was discernable for these three isoforms. The same was true for the two other isoforms of HG.
- **PEDF3308:** It was difficult to discern a clear pattern concerning PEDF3308 and its isoforms.

### Post-translational modifications (aim 2)

Based on the above-mentioned correlation between AT5106 and I_AT111 (albeit by PCA, not traditional bivariate correlation), we decided to focus the biochemical part of the present paper on characterizing some of the post-translational modifications and fragments of AT. The 18 isoforms of AT are highlighted in Fig. 1, and the analyzed isoforms are shown in Table 2. As can be seen in Table 2, we found six truncated forms of AT, and we were able to show that seven isoforms were N-glycosylated. Three of the N-glycosylated isoforms (spots 1605, 1606, and 2601) belonged to AT group V which, as described above, had a large proportion of missing values. However, at least one AT group V isoform was present in seven out of 11 patients compared to two out of 11 healthy controls (*p* = 0.04, Fisher’s exact test).

**Table 2:** Post-translational modifications (PTMs) of 18 isoforms of cerebrospinal fluid alpha-1-antitrypsin

SPOT NUMBER	Mw (kDa)	pI	Unique peptides	Peptides position START-END	PTM
1605	153.0	5.50	18	35–418	N-glycosylation
1606	153.0	5.52	18	35–418	N-glycosylation
2601	153.0	5.54	18	35–418	N-glycosylation
1310	70.9	5.25	14	35–418	–
1308	69.5	5.35	20	35–418	–
2311	69.5	5.39	25	35–418	–
2310	68.2	5.44	25	35–418	–
2308	68.2	5.50	31	35–418	–
216	36.7	4.78	6	180–411	N-terminal truncation
215	35.4	4.80	16	126–418	N-terminal truncation
214	33.4	4.90	6	35–241	C-terminal truncation
111	38.9	5.10	10	126–418	N-terminal truncation
110	37.4	5.15	16	154–418	N-terminal truncation
5106	19.4	6.50	7	299–418	N-terminal truncation
1505	57.6	5.3	12	35–418	N-glycosylation
2504	60.9	5.5	12	35–418	N-glycosylation
2508	68.3	5.5	12	35–418	N-glycosylation
2512	68.2	5.4	12	35–418	N-glycosylation

AT5106 and I_AT111, which were positively inter-correlated in patients by PCA, were both confirmed to be N-terminal truncated fragments. IV_AT1505 was N-glycosylated.

### Regression of clinical pain parameters (aim 3)

First, pain intensity data in patients (“VASPI last week”) was regressed, using the 260 proteins from our earlier study as predictor variables (X-variables). The OPLS model on 11 patients with “VASPI last week” as outcome variable (Y-variable) rendered three components (R^2^ = 0.99, Q^2^ = 0.43), and the results are summarized in Table 3. Notably, the protein having the highest VASPI-VIP (as well as a high and significant Spearman’s rho) was a previously not identified isoform of alpha-1-antitrypsin (spot 2515, Table 3).

**Table 3:** Proteins associated with Visual Analogue Pain Intensity last week (VASPI) in patients with peripheral neuropathic pain

SPOT NB	VASPI-VIP	Protein name	Bivariate correlation with VASPI	
			Spearman’s rho	*p*-value
2515	2.41	Alpha-1-antitrypsin	0.77	0.005*
3308	2.38	Pigment epithelium-derived factor	0.75	0.008*
4204	2.31	Fibrinogen gamma chain	0.72	0.012*
3211	2.14	Pigment epithelium-derived factor	0.58	0.064
7001	2.11	Prostaglandin-H2 D-isomerase	0.63	0.040*
6105	2.01	Kallikrein-6	0.62	0.041*
6104	2.00	Prostaglandin-H2 D-isomerase	−0.54	0.084
1111	1.97	Apolipoprotein E	0.66	0.028*
2405	1.89	Antithrombin	0.65	0.031*
2514	1.87	Beta-Ala-His dipeptidase	0.46	0.163
6613	1.84	Serotransferrin	0.70	0.016*
110	1.83	Alpha-1-antitrypsin	−0.68	0.022*
6206	1.79	Serotransferrin	0.52	0.104
1506	1.78	Beta-Ala-His dipeptidase	0.14	0.678
8004	1.77	Prostaglandin-H2 D-isomerase	−0.26	0.440
7202	1.76	Procollagen C-endopeptidase enhancer 1	0.38	0.244
111	1.75	Alpha-1-antitrypsin	−0.78	0.005*
3409	1.73	Angiotensinogen	0.81	0.003*
6205	1.72	Serotransferrin	−0.45	0.163
8108	1.67	Prostaglandin-H2 D-isomerase	−0.40	0.226
4611	1.66	Hemopexin	0.48	0.140
1205	1.64	Complement factor B	−0.62	0.041*
2205	1.64	Haptoglobin	0.30	0.375
2522	1.63	prothrombin	0.40	0.226
7713	1.62	plasminogen	−0.73	0.103
3302	1.61	Antithrombin	0.55	0.081
2504	1.61	Alpha-1-antitrypsin	0.37	0.257
8414	1.60	IgG heavy chain	−0.44	0.177
6304	1.58	Beta-2-glycoprotein 1	−0.21	0.535
8204	1.57	Prostaglandin-H2 D-isomerase	0.24	0.473
9507	1.56	Complement C4-A	−0.28	0.399
5620	1.55	Serum Albumin	0.42	0.204
3204	1.53	Pigment epithelium-derived factor	0.56	0.075
8413	1.52	IgG heavy chain	−0.23	0.491
7407	1.50	IgG heavy chain	−0.26	0.440

Note: Proteins are listed in decreasing order of importance according to Variable Influence on Projection (VIP) of the OPLS model. Only protein isoforms with VIP ≥ 1.5 are reported (see Methods section). Spot NB refers to the marked protein spot in Additional file 1: Figure S1

**Fig. 3:**
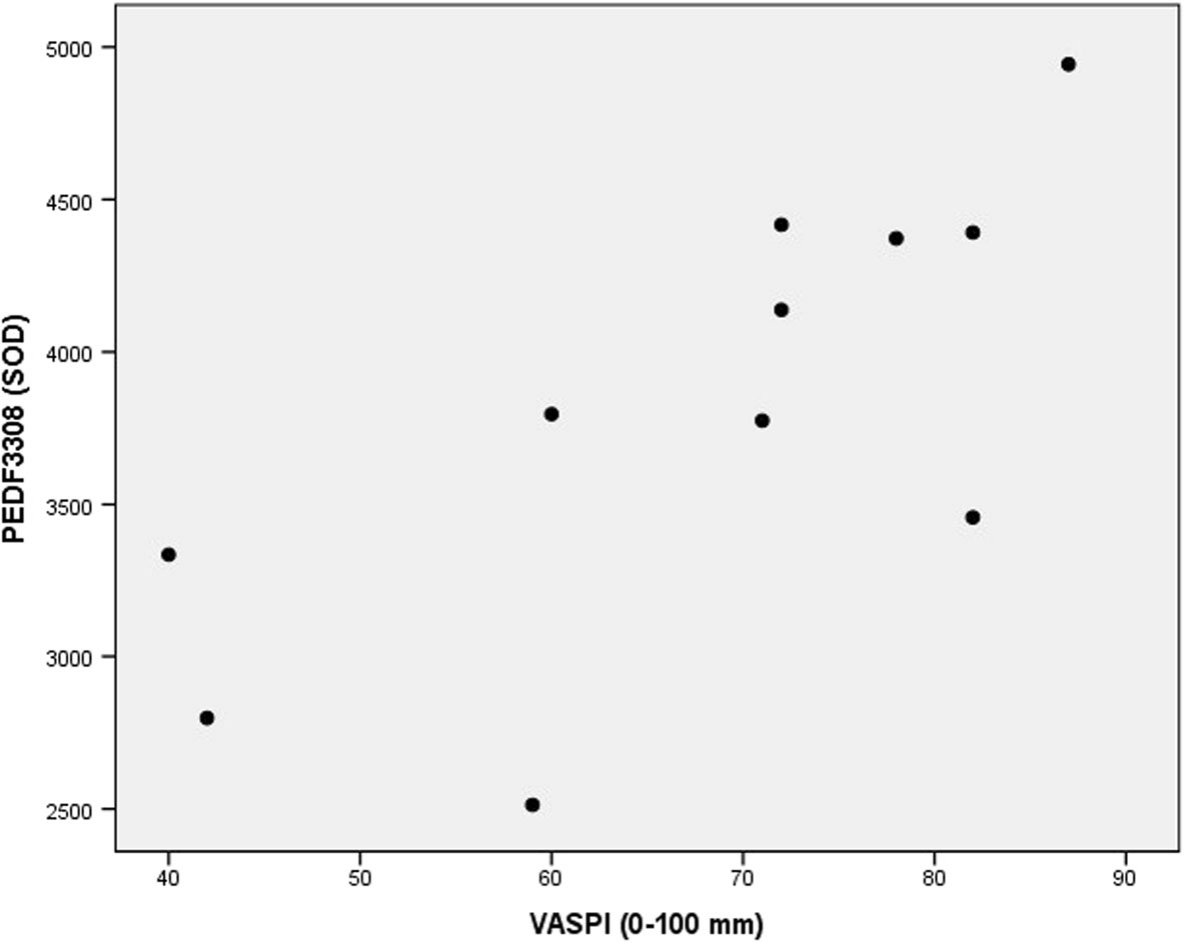
Pain intensity vs PEDF spot 3308. Scatter plot of Visual Analogue Scale Pain Intensity (0–100 mm) last week (VASPI) vs. spot optical density (SOD) of pigment epithelium-derived factor (PEDF) spot 3308 in the cerebrospinal fluid of patients with peripheral neuropathic pain (*n* = 11). Spearman’s rho = 0.75, *p* = 0.008

**Fig. 4:**
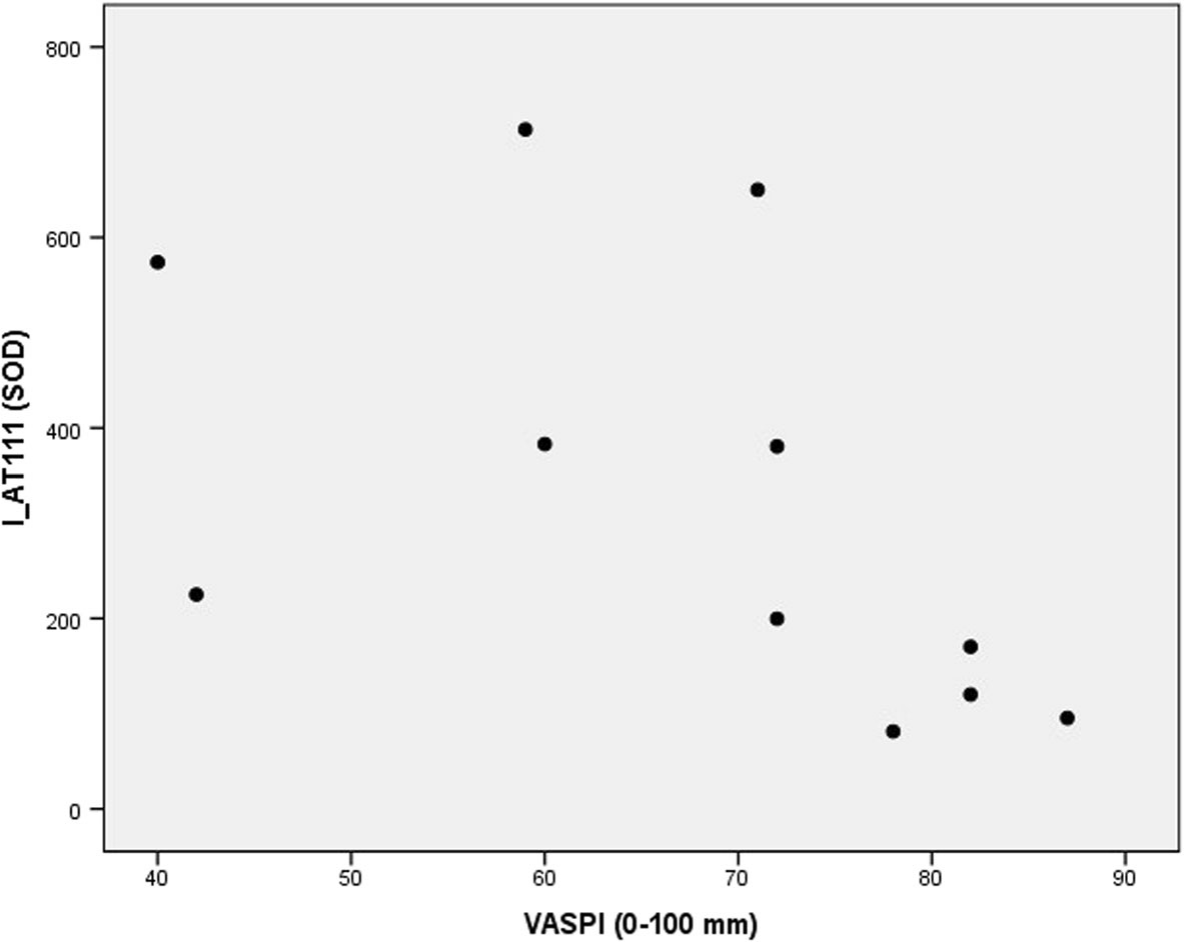
Pain intensity vs AT spot 111. Scatter plot of Visual Analogue Scale Pain Intensity (0–100 mm) last week (VASPI) vs. spot optical density (SOD) of alpha-1-antitrypsin (AT) spot 111 in the cerebrospinal fluid of patients with peripheral neuropathic pain (*n* = 11). Spearman’s rho = − 0.781, *p* = 0.005

**Fig. 5:**
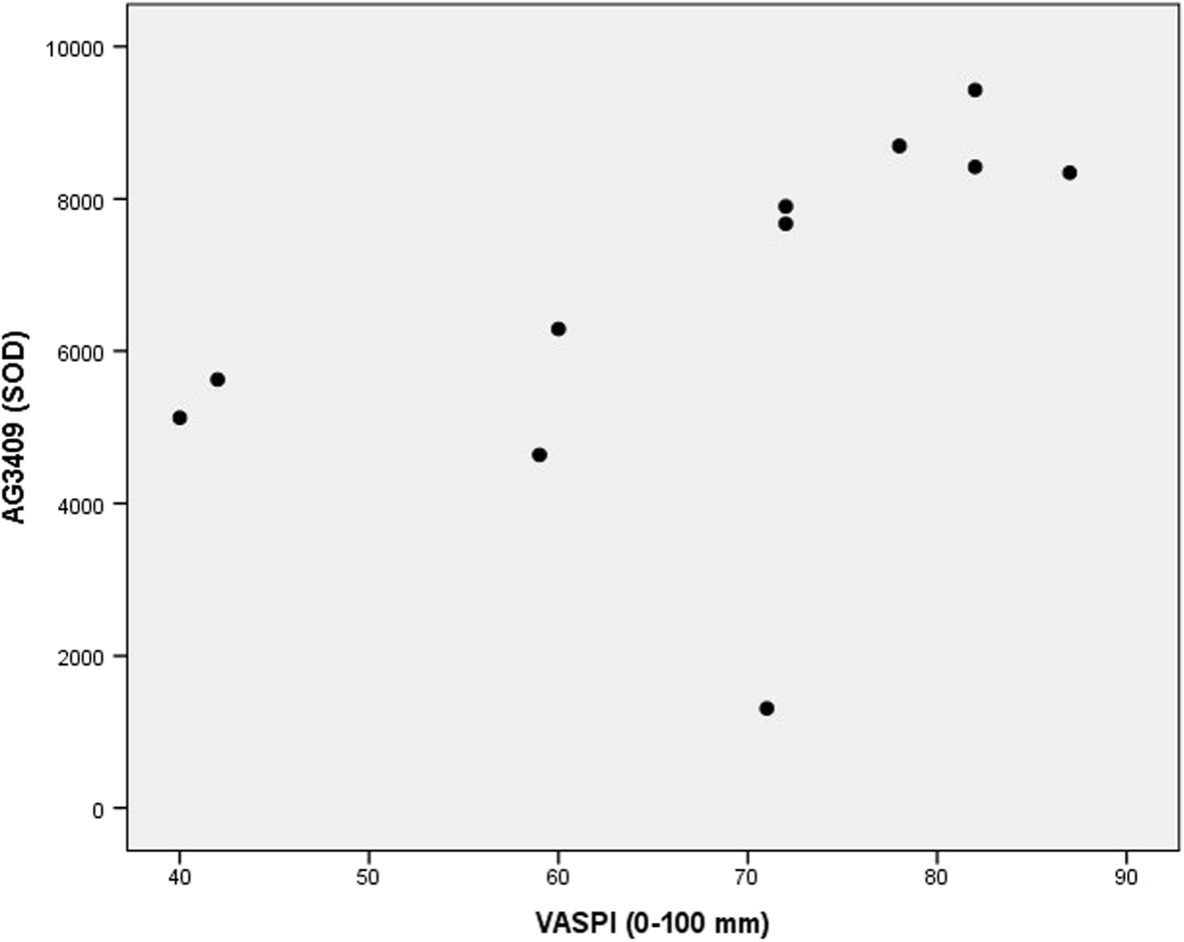
Pain intensity vs AG spot 3409. Scatter plot of Visual Analogue Scale Pain Intensity (0–100 mm) last week (VASPI) vs. spot optical density (SOD) of angiotensinogen (AG) spot 3409 in the cerebrospinal fluid of patients with peripheral neuropathic pain (*n* = 11). Spearman’s rho = 0.81, *p* = 0.003

Of the proteins described in Aim 1 (including Table 2), the following four proteins had a high VIP (i.e. VIP≥ 1.5) *and* a significant Spearman’s rho correlation coefficient:

- PEDF3308 had VIP = 2.38, which was the second-highest VIP of the model (rank 2 out of 260 proteins). The bivariate correlation between “VASPI last week” and PEDF3308 was positive (rho = 0.75, *p* = 0.008), Fig. 3.
- I_AT110 had VIP = 1.83 (rank 13 out of 260 proteins). The bivariate correlation between “VASPI last week” and I_AT110 was negative (rho = − 0.676, *p* = 0.022). Going back to Fig. 2b, it can be seen that I_AT110 positively inter-correlated with I_AT111 (and hence with AT5106) in patients, and this was confirmed by classical bivariate correlation (rho = 0.664, *p* = 0.026); in healthy controls, no such correlation existed between I_AT110 and I_AT111 (rho = 0.191, *p* = 0.574).
- I_AT111 had VIP = 1.75 (rank 19 out of 260 proteins). The bivariate correlation between “VASPI last week” and I_AT111 was negative (rho = − 0.781, *p* = 0.005), Fig. 4.
- AG3409 had VIP = 1.73 (rank 20 out of 260 proteins). The bivariate correlation between “VASPI last week” and AG3409 was positive (rho = 0.81, *p* = 0.003), Fig. 5.

Moreover, PEDF3211, which was one of the five isoforms of PEDF in Aim 1 above, also had a very high VASPI-VIP (VIP = 2.14, rank 4 out of 260 proteins), albeit with a non-significant Spearman’s rho (rho = 0.58, *p* = 0.064).

Hence, three isoforms of AT, two isoforms of PEDF, and one isoform of angiotensinogen “reappeared” as major results when regressing VASPI, i.e., they were major findings both in the present study and in our previous study [16].

Then, pain duration in patients was regressed using the 260 proteins from our earlier study as predictor variables (X-variables). The OPLS model on 11 patients with “pain duration” as outcome variable (Y-variable) had 3 components (R^2^ = 0.99, Q^2^ = 0.54), and the results are summarized in Table 4. Of the proteins described above in Aim 1 (including Table 2), seven proteins had a high VIP for pain duration (i.e. VIP≥1.5), but none of these proteins had a significant Spearman’s rho correlation coefficient for pain duration. Among these seven proteins, however, the presence of AG3409 was noted, as it was rather highly ranked among the 260 proteins (VIP = 1.95, rank 12 and rho = − 0.39, *p* = 0.233).

**Table 4:** Proteins associated with pain duration in patients with peripheral neuropathic pain

SPOT NB	Pain duration-VIP	Protein name	Bivariate correlation with pain duration	
			Spearman’s rho	*p*-value
2312	2.31	Zinc-Alpha-2-glycoprotein	0.50	0.120
7712	2.27	Plasminogen	0.82	0.023*
1205	2.26	Complement factor B	0.78	0.005*
7711	2.23	Plasminogen	0.61	0.148
3502	2.16	Hemopexin	0.55	0.082
3309	2.15	Haptoglobin	0.29	0.392
4309	2.14	Haptoglobin	0.71	0.071
2106	2.09	Apolipoprotein E	0.55	0.079
206	2.07	Haptoglobin	0.77	0.072
2601	2.05	Alpha-1-antitrypsin	0.89	0.019*
3106	2.03	Clusterin	−0.67	0.024*
3409	1.95	Angiotensinogen	−0.39	0.233
409	1.93	Alpha-2-HS-glycoprotein	0.46	0.159
1203	1.86	Haptoglobin	0.52	0.105
7713	1.86	Plasminogen	0.77	0.072
6001	1.82	Alpha-1-antitrypsin	−0.54	0.091
2311	1.82	Alpha-1-antitrypsin	0.38	0.245
6611	1.75	Serotransferrin	−0.57	0.067
6614	1.73	Serotransferrin	−0.58	0.062
6610	1.72	Serotransferrin	−0.67	0.023*
3111	1.71	Prostaglandin-H2 D-isomerase	−0.54	0.085
412	1.69	Alpha-2-HS-glycoprotein	0.44	0.179
6609	1.67	Serotransferrin	−0.72	0.013*
1211	1.67	Haptoglobin	0.30	0.377
3210	1.66	Haptoglobin	0.52	0.098
3010	1.66	Tetranectin	−0.21	0.527
2310	1.63	Alpha-1-antitrypsin	0.25	0.466
7403	1.61	Complement C3	0.59	0.055
4105	1.61	Prostaglandin-H2 D-isomerase	−0.51	0.113
5306	1.59	Pigment epithelium-derived factor	−0.60	0.050
2108	1.57	Apolipoprotein E	−0.56	0.073
208	1.56	Haptoglobin	0.35	0.290
8001	1.55	Phosphatidylethanolamine binding protein	−0.46	0.154
1111	1.53	Apolipoprotein E	−0.55	0.082

Note: Proteins are listed in decreasing order of importance according to Variable Influence on Projection (VIP) of the OPLS model. Only protein isoforms with VIP ≥ 1.5 are reported (see Methods section). Spot NB refers to the marked protein spot in Additional file 1: Figure S1

## Discussion

The results presented in this paper suggest that fragments of AT might be considered as potential biomarkers for pathophysiological processes in the spinal cord of patients suffering from chronic peripheral neuropathic pain. AT in CSF is considered to be plasma-derived [28], but exposure to pathological processes in the central nervous system during diffusion from plasma to CSF could potentially lead to a disease-specific fragmentation process detectable in the CSF. However, local CNS production of AT in pathological conditions is also a possibility [29, 30].

The results presented here should be viewed as hypothesis-generating [31], and the low number of subjects in the study is of course a strong limitation, as is the age difference between the groups. Another limitation is the fact that the patients were using analgesics, introducing a potential confounding effect; moreover, concerning paracetamol and Non-Steroidal Anti-Inflammatory Drugs (NSAID), the percentages reported in Table 1 might perhaps somewhat underestimate the size of this problem because treatment “as needed” was not recorded. The difficulties inherent in CSF sampling (not least in pain patients) should however be remembered, and the usefulness of the CSF for CNS biomarker studies should be emphasized [13, 32]. Human pain proteomic CSF studies that actually report biomarker candidates are rare, and those that have been published typically report about 10 subjects per group [33, 34].

Protein fragments are emerging as important potential biomarkers in medicine in general [11]. Indeed, in the context of dementia research, the term “protein fragmentology” has been used [12]. What makes protein fragments so interesting in a CNS context is that their small size could enable them to cross the BBB easier than full-length proteins, and they would theoretically therefore be easier to detect in plasma [12]. As taking a blood sample is much easier than doing a lumbar puncture for CSF analysis, this is a very important practical aspect to take into consideration when searching for useable biomarkers.

Three fragments of AT stand out as especially interesting: AT5106, I_AT111, and I_AT110. AT5106 is a very small fragment (Table 2 and Fig. 1), and in our previous study it had the second-highest discriminatory power between groups, being down-regulated in patients [16]. The correlation of AT5106 with I_AT111 in patients (as revealed by comparing the PCA column loading plots, Fig. 2a and b), lead to a particular interest also in the latter fragment. Indeed, I_AT111 turned out to be an important predictor of VASPI by MVDA, and it correlated negatively with VASPI by traditional bivariate statistics (Fig. 4). In our previous study, I_AT111 was shown to be up-regulated in patients [16]. We therefore speculate that the combination of down-regulated N-truncated AT5106 and up-regulated N-truncated I_AT111 could mirror disease-specific processes in the spinal cord. The negative correlation with VASPI could perhaps indicate that I_AT111 indirectly mirrors the efficacy of anti-nociceptive mechanisms, i.e. it would be up-regulated in patients, and those who have more of it would have less activity in the nociceptive pathways. Does I_AT111 hence indirectly mirror an anti-inflammatory compensating mechanism in the spinal cord? The speculative nature of this line of reasoning must be emphasized. By PCA as well as by traditional bivariate correlation, it is also notable that I_AT111 and I_AT110 positively inter-correlated in patients but not in healthy controls. All in all, we speculate that the interactions of these three fragments of AT might mirror disease-specific processes in the spinal cord.

Isoform IV_AT1505, which was one of the seven most discriminating proteins in our earlier study [16], did not contribute to the PCA models of Aim 1 and did not appear as a result of Aim 3.

Turning to glycosylated isoforms of AT, subgroup V (consisting of V_AT1605, V_AT1606, and V_AT2601, Fig. 1) appears interesting. It is true that these isoforms have a high percentage of missing values (in 68%, 68% and 63% of cases, respectively), but the distribution of glycosylated isoforms of group V differed between groups, the presence of glycosylated isoforms in group V being associated with the patients group. Indeed, it has been said that the pattern of AT glycosylation can be an indicator of the immune modulatory properties of AT [35]. The drawback of “big” glycosylated isoforms, as compared to protein fragments, is their relatively low ability to cross the BBB and hence lower probability to be detectable in plasma. All in all, we think that fragments and/or glycosylated isoforms of AT seem to have “biomarker potential” in pain medicine. Further studies, both in CSF and plasma [35], seem warranted.

Although we chose to focus on AT isoforms in the present study, future work on the isoforms of AG and PEDF would be interesting. Concerning AG3409, which had the highest discriminative power between groups in our previous study [16], it is notable that it reappears in the results of Aim 3 in the present paper (Fig. 5). Although this of course might be a false positive finding, it is nonetheless interesting that the same protein reappears when regressing clinical parameters in the patients group. Hence, AG3409 discriminated between patients and healthy controls [16], but was *also* positively correlated to VASPI (and had a high VIP when regressing pain duration). The renin-angiotensin system seems to be involved in nociception processing [36, 37], and is a potential pain therapeutic target [38–40]; investigating this particular isoform seems to be an important line of future work. Does this isoform mirror pro-nociceptive activity in the spinal cord of patients with neuropathic pain?

PEDF3308 was down-regulated in patients in our previous study [16]. In the present study, this isoform also reappears in the results of Aim 3, and this even more forcefully than AG3409 as PEDF3308 had the *second-highest* VIP of the model (rank 2 out of 260 proteins). PEDF3308 correlated positively with VASPI (Fig. 3). PEDF protects against glutamate-caused excitotoxicity [41], and we therefore speculate that our findings could indicate a direct anti-nociceptive activity of PEDF3308, which would be “consumed” in patients (hence down-regulated and at the same time positively correlated to VASPI – those “consuming” more of it having less pain). This is of course extremely speculative, but seems to make physiological sense.

Going back to AT, one might wonder why such a well-known protein would be a specific biomarker for a pathological pain condition. In this context, it is important to remember that PTMs are very important physiologically. PTMs modulate enzyme activity, protein turnover and localization, protein-protein interactions, various signaling cascades, DNA repair, and cell division [5]. It is becoming increasingly clear that PTMs are important in both health and disease. For instance, posttranslational glycosylation patterns are said to be an extremely sensitive indicator of intracellular conditions, and the fields of glycoproteomics is emerging as an important contributor in the search for biomarkers in different medical conditions [42]. Hence, PTM-patterns are probably important when trying to identify the molecular “fingerprints” of different pain conditions. Other important forms of PTMs include acetylation, deamidation, hydroxylation, nitration, palmitoylation, phosphorylation, sulfation and ubiquitination [5, 43]. Therefore, looking only at *total* levels of a particular protein is probably often too simplistic, and an “old” and well-known protein like AT might very well, due to PTMs, mirror disease-specific processes. The familiarity of AT should not make one a priori consider it uninteresting as a biomarker.

In the words of Pavlou et al., we have studied “a small number of samples from diseased and nondiseased groups” in order to “identify molecules exhibiting discriminating potential” [17]. To correctly evaluate our findings, it is important to understand that the present study was not intended to generate clinical biomarker candidates. If that had been our purpose, dozens or perhaps hundreds of samples would have been necessary. Instead, using the terminology proposed by Pavlou et al., this was an early discovery phase, pre-clinical exploratory study [17]. For such studies, in which the aim is to strive towards a better understanding of molecular pathology in humans, the study design requirements are different from clinical biomarker studies [20].

## Conclusions

On the basis of the findings reported in the present paper, we present the hypothesis that fragments and/or glycosylated isoforms of alpha-1-antitrypsin might be considered as potential biomarkers of the pathophysiological processes in the spinal cord of neuropathic pain patients. The biomarker potential of protein fragments should be taken into account by pain researchers. Biomarkers with high specificity and sensitivity are difficult to find, and the combinatorial power of a panel of different biomarkers has been suggested as a solution this problem [44]. This is in line with modern systems biology [45], the focus lying not on a particular “magic bullet” protein but on networks of mutually interacting proteins. In such a context, the above-mentioned combination of down-regulated N-truncated AT5106 and up-regulated N-truncated I_AT111 could perhaps be of value. More research is needed, both in CSF and plasma, in order to perhaps confirm this hypothesis.

## Additional file

Additional file 1: **Figure S1.** Two dimensional gel electrophoregram of CSF proteins. The marked spot numbers refer to the identified proteins in Tables 3 and 4. (JPG 76 kb)

## Abbreviations

AG: Angiotensinogen
AT: Alpha-1-antitrypsin
BBB: Blood-brain barrier
CNS: Central nervous system
CSF: Cerebrospinal fluid
HG: Haptoglobin
MVDA: Multivariate data analysis by projection
OPLS: Orthogonal partial least squares projection to latent structures
PC: Principal component
PCA: Principal component analysis
PEDF: Pigment epithelium-derived factor
PTM: Post-translational modification
SOD: Spot optical density
VASPI: Visual analogue scale pain intensity
VIP: Variable influence on projection

## References

[1] Mao J (2009). Translational pain research: achievements and challenges. J Pain.

[2] Borsook D, Becerra L, Hargreaves R (2011). Biomarkers for chronic pain and analgesia. Part 1: the need, reality, challenges, and solutions. Discov Med.

[3] Internat (2004). Human Genome Sequencing Consortium. Finishing the euchromatic sequence of the human genome. Nature.

[4] Jensen ON (2004). Modification-specific proteomics: characterization of post-translational modifications by mass spectrometry. Curr Opin Chem Biol.

[5] Karve TM, Cheema AK (2011). Small changes huge impact: the role of protein posttranslational modifications in cellular homeostasis and disease. J Amino Acids.

[6] Kim MS, Pinto SM, Getnet D, Nirujogi RS, Manda SS, Chaerkady R, Madugundu AK, Kelkar DS, Isserlin R, Jain S (2014). A draft map of the human proteome. Nature.

[7] Niederberger E, Geisslinger G (2008). Proteomics in neuropathic pain research. Anesthesiology.

[8] Niederberger E, Kuhlein H, Geisslinger G (2008). Update on the pathobiology of neuropathic pain. Expert Rev Proteomics.

[9] Pan S, Chen R, Aebersold R, Brentnall TA (2011). Mass spectrometry based glycoproteomics--from a proteomics perspective. Mol Cell Proteomics.

[10] Drake PM, Cho W, Li B, Prakobphol A, Johansen E, Anderson NL, Regnier FE, Gibson BW, Fisher SJ (2010). Sweetening the pot: adding glycosylation to the biomarker discovery equation. Clin Chem.

[11] Genovese F, Karsdal MA (2015). Protein degradation fragments as diagnostic and prognostic biomarkers of connective tissue diseases: understanding the extracellular matrix message and implication for current and future serological biomarkers. Expert Rev Proteomics.

[12] Inekci D, Jonesco DS, Kennard S, Karsdal MA, Henriksen K (2015). The potential of pathological protein fragmentation in blood-based biomarker development for dementia - with emphasis on Alzheimer's disease. Front Neurol.

[13] Lai ZW, Petrera A, Schilling O (2015). The emerging role of the peptidome in biomarker discovery and degradome profiling. Biol Chem.

[14] Carlsson-Jonsson A, Gao T, Hao JX, Fransson R, Sandstrom A, Nyberg F, Wiesenfeld-Hallin Z, Xu XJ (2014). N-terminal truncations of substance P 1–7 amide affect its action on spinal cord injury-induced mechanical allodynia in rats. Eur J Pharmacol.

[15] Jensen TS, Baron R, Haanpaa M, Kalso E, Loeser JD, Rice AS, Treede RD (2011). A new definition of neuropathic pain. Pain.

[16] Bäckryd E, Ghafouri B, Carlsson AK, Olausson P, Gerdle B (2015). Multivariate proteomic analysis of the cerebrospinal fluid of patients with peripheral neuropathic pain and healthy controls - a hypothesis-generating pilot study. J Pain Res.

[17] Pavlou MP, Diamandis EP, Blasutig IM (2013). The long journey of cancer biomarkers from the bench to the clinic. Clin Chem.

[18] Eriksson L, Byrne T, Johansson E, Trygg J, Vikström C (2013). Multi- and Megavariate data analysis: basic principles and applications.

[19] Wheelock AM, Wheelock CE (2013). Trials and tribulations of 'omics data analysis: assessing quality of SIMCA-based multivariate models using examples from pulmonary medicine. Mol BioSyst.

[20] Mischak H, Vlahou A, Righetti PG, Calvete JJ (2014). Putting value in biomarker research and reporting. J Proteome.

[21] Bäckryd E, Sorensen J, Gerdle B (2015). Ziconotide trialing by Intrathecal bolus injections: an open-label non-randomized clinical trial in postoperative/posttraumatic neuropathic pain patients refractory to conventional treatment. Neuromodulation.

[22] Dworkin RH, Turk DC, Farrar JT, Haythornthwaite JA, Jensen MP, Katz NP, Kerns RD, Stucki G, Allen RR, Bellamy N (2005). Core outcome measures for chronic pain clinical trials: IMMPACT recommendations. Pain.

[23] Treede RD, Jensen TS, Campbell JN, Cruccu G, Dostrovsky JO, Griffin JW, Hansson P, Hughes R, Nurmikko T, Serra J (2008). Neuropathic pain: redefinition and a grading system for clinical and research purposes. Neurology.

[24] Zigmond AS, Snaith RP (1983). The hospital anxiety and depression scale. Acta Psychiatr Scand.

[25] Sullivan MJ, Bishop SR, Pivik J (1995). The pain catastrophizing scale: development and validation. Psychol Assess.

[26] Sullivan M, Karlsson J, Ware JE (1995). The Swedish SF-36 health survey—I. Evaluation of data quality, scaling assumptions, reliability and construct validity across general populations in Sweden. Soc Sci Med.

[27] Ghafouri B, Irander K, Lindbom J, Tagesson C, Lindahl M (2006). Comparative proteomics of nasal fluid in seasonal allergic rhinitis. J Proteome Res.

[28] Irani DN, Irani DN (2009). Properties and Composition of Normal Cerebrospinal Fluid. Cerebrospinal Fluid in Clinical Practice.

[29] Jesse S, Lehnert S, Jahn O, Parnetti L, Soininen H, Herukka SK, Steinacker P, Tawfik S, Tumani H, von Arnim CA (2012). Differential sialylation of serpin A1 in the early diagnosis of Parkinson's disease dementia. PLoS One.

[30] Gollin PA, Kalaria RN, Eikelenboom P, Rozemuller A, Perry G (1992). Alpha 1-antitrypsin and alpha 1-antichymotrypsin are in the lesions of Alzheimer's disease. Neuroreport.

[31] Biesecker LG (2013). Hypothesis-generating research and predictive medicine. Genome Res.

[32] Roche S, Gabelle A, Lehmann S (2008). Clinical proteomics of the cerebrospinal fluid: towards the discovery of new biomarkers. Proteomics Clin Appl.

[33] Conti A, Ricchiuto P, Iannaccone S, Sferrazza B, Cattaneo A, Bachi A, Reggiani A, Beltramo M, Alessio M (2005). Pigment epithelium-derived factor is differentially expressed in peripheral neuropathies. Proteomics.

[34] Liu XD, Zeng BF, Xu JG, Zhu HB, Xia QC (2006). Proteomic analysis of the cerebrospinal fluid of patients with lumbar disk herniation. Proteomics.

[35] McCarthy C, Saldova R, Wormald MR, Rudd PM, McElvaney NG, Reeves EP (2014). The role and importance of glycosylation of acute phase proteins with focus on alpha-1 antitrypsin in acute and chronic inflammatory conditions. J Proteome Res.

[36] Nemoto W, Nakagawasai O, Yaoita F, Kanno S, Yomogida S, Ishikawa M, Tadano T, Tan-No K (2013). Angiotensin II produces nociceptive behavior through spinal AT1 receptor-mediated p38 mitogen-activated protein kinase activation in mice. Mol Pain.

[37] Smith MT, Lau T, Wallace VC, Wyse BD, Rice AS (2014). Analgesic efficacy of small-molecule angiotensin II type 2 receptor antagonists in a rat model of antiretroviral toxic polyneuropathy. Behav Pharmacol.

[38] Finnerup NB, Baastrup C (2014). Angiotensin II: from blood pressure to pain control. Lancet.

[39] Rice AS, Dworkin RH, McCarthy TD, Anand P, Bountra C, McCloud PI, Hill J, Cutter G, Kitson G, Desem N (2014). EMA401, an orally administered highly selective angiotensin II type 2 receptor antagonist, as a novel treatment for postherpetic neuralgia: a randomised, double-blind, placebo-controlled phase 2 clinical trial. Lancet.

[40] Smith MT, Muralidharan A (2015). Targeting angiotensin II type 2 receptor pathways to treat neuropathic pain and inflammatory pain. Expert Opin Ther Targets.

[41] Craword SE, Fitchev P, Veliceasa D, Volpert OV (2013). The many facets of PEDF in drug discovery and disease: a diamond in the rough or split personality disorder?. Expert Opin Drug Discov.

[42] Hua S, An HJ (2012). Glycoscience aids in biomarker discovery. BMB Rep.

[43] Rogowska-Wrzesinska A, Le Bihan MC, Thaysen-Andersen M, Roepstorff P (2013). 2D gels still have a niche in proteomics. J Proteome.

[44] Drucker E, Krapfenbauer K (2013). Pitfalls and limitations in translation from biomarker discovery to clinical utility in predictive and personalised medicine. EPMA J.

[45] Antunes-Martins A, Perkins JR, Lees J, Hildebrandt T, Orengo C, Bennett DL (2013). Systems biology approaches to finding novel pain mediators. Wiley Interdiscip Rev Syst Biol Med.

