# Supplementary figures and images for: Do fragments and glycosylated isoforms of alpha-1-antitrypsin in CSF mirror spinal pathophysiological mechanisms in chronic peripheral neuropathic pain? An exploratory, discovery phase study
Source: BMC Neurol. 2018 Aug 16;18:116. doi: 10.1186/s12883-018-1116-2 (PMC6097305; doi:10.1186/s12883-018-1116-2)

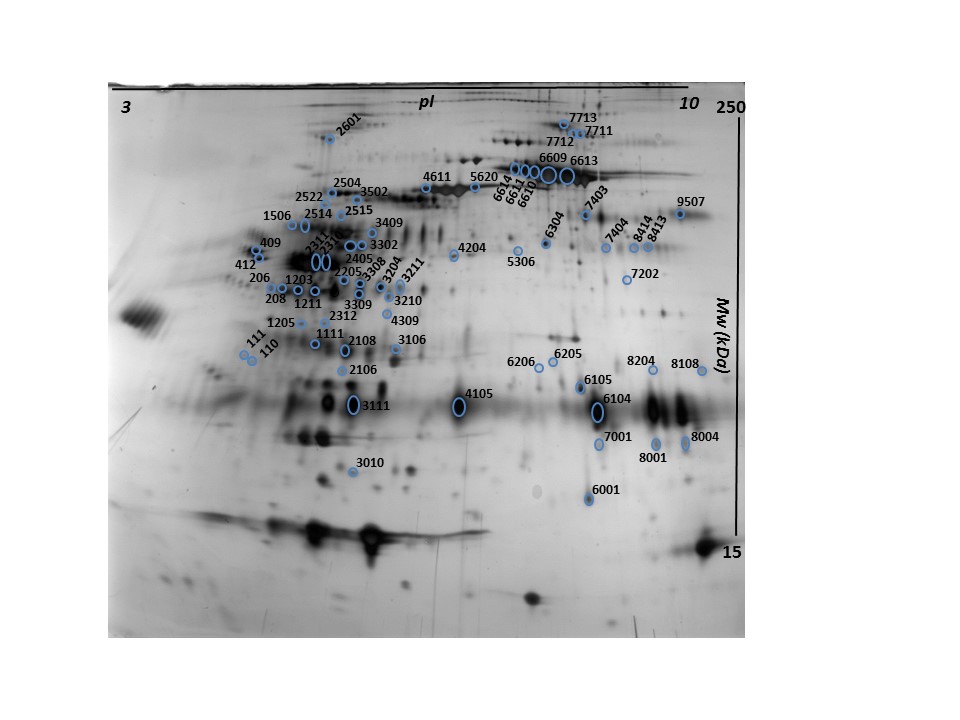

Supplement: Supplementary file 1 — Figure S1. Two dimensional gel electrophoregram of CSF proteins. The marked spot numbers refer to the identified proteins in Tables 3 and 4. (JPG 76 kb) [file 12883_2018_1116_MOESM1_ESM.jpg]
